# Does ‘Dry Hit’ vaping of vitamin E acetate contribute to EVALI? Simulating toxic ketene formation during e-cigarette use

**DOI:** 10.1371/journal.pone.0238140

**Published:** 2020-09-03

**Authors:** Milad Narimani, Gabriel da Silva

**Affiliations:** Department of Chemical Engineering, The University of Melbourne, Melbourne, Victoria, Australia; Pandit Deendayal Petroleum University, India, INDIA

## Abstract

Vitamin E acetate (VEA) is strongly linked to the outbreak of electronic-cigarette or vaping product use-associated lung injury (EVALI). It has been proposed that VEA decomposition to ketene–a respiratory poison that damages lungs at low ppm levels–may play a role in EVALI. However, there is no information available on the temperature at which VEA decomposes and how this correlates with the vaping process. We have studied the temperature-dependent kinetics of VEA decomposition using quantum chemical and statistical mechanical modelling techniques, developing a chemical kinetic model of the vaping process. This model predicts that, under typical vaping conditions, the use of VEA contaminated e-cigarette products is unlikely to produce ketene at harmful levels. However, at the high temperatures encountered at low e-cigarette product levels, which produce ‘dry hits’, ketene concentrations are predicted to reach acutely toxic levels in the lungs (as high as 30 ppm). We therefore hypothesize that dry hit vaping of e-cigarette products containing VEA contributes to EVALI.

## Introduction

The rapidly growing number of clinically reported cases of e-cigarette, or vaping, product use-associated lung injury (EVALI) since August 2019 has placed this as a major public health concern in the United States [1–4]. The US Centers for Disease Control and Prevention (CDC) reported on the 14^th^ of January 2020 that at least 2,668 hospitalized EVALI patients and 60 deaths had been recorded across the country [5]. All EVALI patients reported a history of using e-cigarette or vaping products in the 3 months preceding symptom onset, which suggests new or increased exposure to one or more toxicants from the use of e-cigarette products.

**Fig 1:**
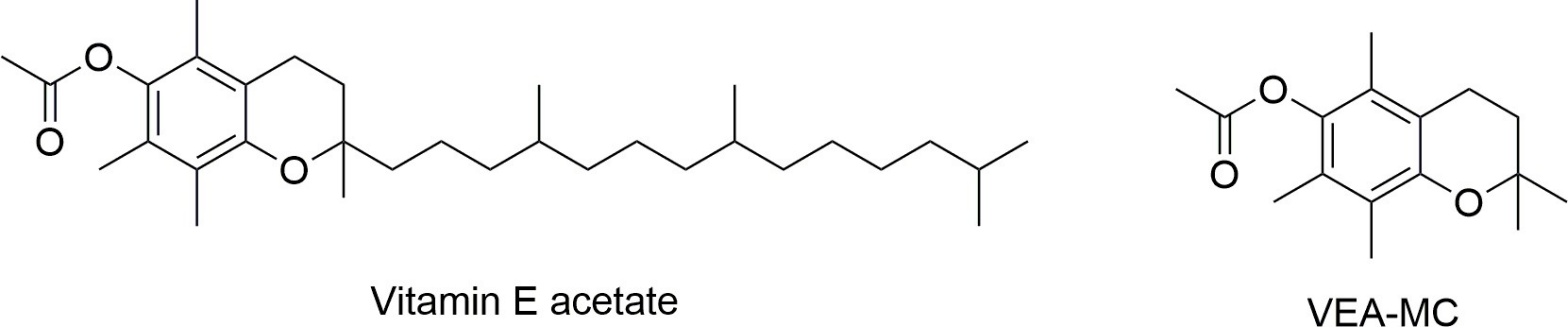
Chemical structure of vitamin E acetate and the studied model compound VEA-MC.

Analysis of bronchoalveolar lavage fluid samples from hospitalized EVALI cases and healthy people identified vitamin E acetate (VEA, Fig 1) as a common toxicant in the lungs of many EVALI patients [2, 6–8]. The addition of vitamin E acetate as a thickening agent or diluent to product fluid began to appear in the illicit market in late 2018 or early 2019 and gained popularity in 2019. Although not definitively proven, the link between EVALI and vitamin E acetate is strong, and much of the associated research has now turned to examining this compound.

As well as being a toxicant in its own right, there is the potential that vaping pyrolysis of vitamin E acetate produces toxic gases which can escalate pulmonary injuries [9]. Of particular concern is ketene (CH_2_CO), which is acutely toxic and immediately dangerous to life or health when inhaled at concentrations of around 5 parts per million (ppm) [10]. However, the ability for vitamin E acetate to decompose to ketene and other toxicants will strongly depend on the working temperature of the vaping device. Although ketene has been implicated as a thermal decomposition product of vitamin E acetate [9], the temperature at which it is evolved under representative vaping conditions remains unknown. The boiling point of VEA is around 220°C [11], whereas e-cigarettes typically operate at around 170°C in wet-through-wick conditions [12, 13]. Heating coil temperatures, however, can be highly variable, and strongly depend on the e-liquid fill level and composition. For instance, working temperatures as high as 1008°C have been measured under dry-through-wick conditions [13].

In this study, the detailed mechanism and kinetics of vitamin E acetate decomposition is determined using computational chemistry and statistical reaction rate theory techniques applied to a VEA model compound (VEA-MC, Fig 1). This model compound has a truncated side-chain which permits higher-level ab initio calculations without altering the reaction mechanism. The obtained kinetic parameters are used to simulate vitamin E acetate pyrolysis across the temperature range relevant to e-cigarettes. This work demonstrates that gas-phase decomposition to ketene is only predicted to be significant at the higher range of e-cigarette operating temperatures, but that these dry hits can potentially deliver ketene at thousands of ppm.

## Methods

Electronic structure theory calculations were carried out in the Gaussian 16 and ORCA program suites [14, 15]. Geometry optimizations and vibrational frequency calculations of stationary points were performed at the M06-2X/6-31G(2df,p) level of theory. Transition state connectivity to reactants and products was verified using intrinsic reaction coordinate scans. Thereafter, single point energy calculations were carried out on optimized structures using the dispersion corrected double hybrid functional DSD-PBEB95-D3BJ in conjunction with the triple zeta basis set def2-TZVPP. This double hybrid model chemistry yields a low mean absolute deviation (MAD) of around 2.3 kcal/mol for barrier height calculations, at moderate computational cost [16, 17]. The DSD-PBEB95-D3BJ/def2-TZVPP energies are compared to the M06-2X results in the S1 File and the mean difference in barrier heights is 2.9 kcal/mol.

Rate coefficient calculations were performed according to canonical transition state theory. Calculations were carried out in the Multiwell-2016 program suite using the M06-2X/6-31G(2df,p) structures and vibrational frequencies with the DSD-PBEB95-D3BJ/def2-TZVPP electronic energies. These parameters are listed in the S1 File. Calculated rate coefficients from 300 ‒ 2000 K were fit to Arrhenius expressions and used to form a chemical kinetic model of the VEA vaping process. Thermodynamic properties were derived from the M06-2X/6-31G(2df,p) calculation results according to standard statistical mechanical formulae, with 298 K enthalpies of formation estimated from group additivity. The kinetic and thermodynamic model files are included as S1 File.

The CHEMKIN-PRO program suite was utilized to simulate product formation during the vaping pyrolysis of VEA from 100 to 1000°C. The heating section of the e-cigarette was treated as a cylindrical plug flow reactor of 20 mm length and 2 mm internal diameter. The reactor was set to work adiabatically at a pressure of 1 atm, which resulted in residence times of 29 to 8 ms across the studied temperature range.

## Results and discussion

**Fig 2:**
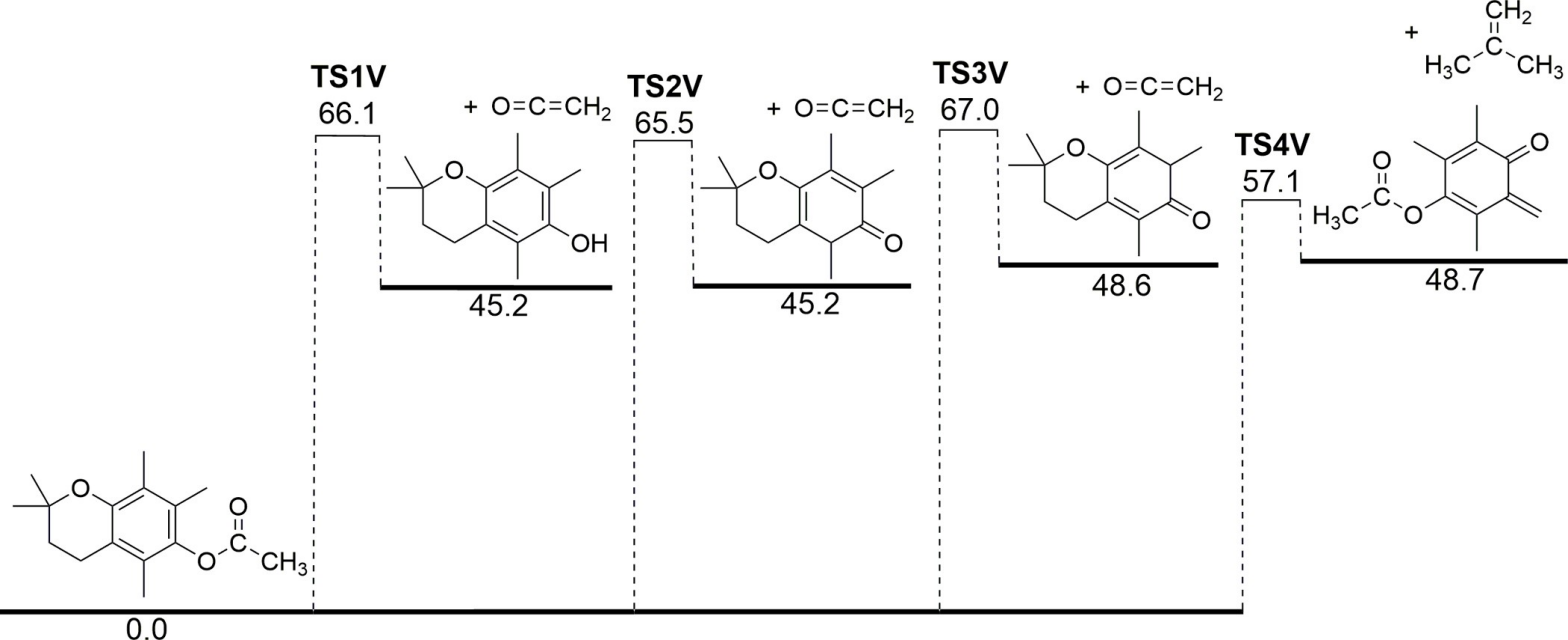
Potential energy diagram for the thermal decomposition of a vitamin E acetate model compound, VEA-MC. Energies (0 K enthalpies, kcal mol^-1^) are calculated at the DSD-PBEB95-D3(BJ)/def2-TZVPP level of theory.

We find that the first stage of VEA decomposition proceeds through four different channels, as illustrated in Fig 2 for the model compound VEA-MC. Three competitive pathways for ketene release are identified, with the lowest-energy reaction involving concerted C—O bond cleavage and 1,3-H atom shift from the methyl group to the ring-bound O atom of the acetyl moiety (**TS1V**). This reaction was identified by Wu and O’Shea [9], and proceeds with a barrier height of 66.1 kcal/mol and releases vitamin E (or chromanol in the case of the model compound) along with ketene. Alternatively, 1,4-H shifts from the methyl group onto the aromatic ring can form ketene along with the keto tautomers of vitamin E, with barrier heights of 65.5 kcal/mol (**TS2V**) and 67.0 kcal/mol (**TS3V**).

The most energetically favorable pathway for VEA decomposition does not involve ketene formation at all. Instead, a retro-Diels Alder reaction (**TS4V**) produces trimethyl quinone methide acetate (TQMA), along with elimination of the ethylene-bound hydrocarbon side-chain (in the model compound this is isobutylene). The barrier height for this process, at 57.1 kcal/mol, is considerably lower than those leading to the direct production of ketene. This reaction mechanism is common to the chroman functional group [18, 19], and has been proposed as the dominant initial step in VEA decomposition [9]. For this key reaction we tested the effect that the aliphatic chain length in the model compound has on the barrier height, with the results shown in S1 Fig in S1 File. There is a decrease in the barrier height of around 0.5 kcal/mol as the chain length is increased from 1 to 2 C atoms. Beyond this there is negligible change in the barrier heights, up to 5 carbon atoms (the largest computationally feasible structure). Given the change in barrier height with chain length is considerably less than the expected error in the model chemistry, we are confident that VEA-MC is an appropriate surrogate for VEA. Subsequently, we therefore assume that VEA decomposes with the same kinetic parameters as are determined for VEA-MC.

Rate coefficients (*k*, s^-1^) for the decomposition of VEA-MC were calculated for all four reactions shown in Fig 2, across the temperature range of 300–2000 K. The reactions exhibit classical Arrhenius behavior (S2 Fig in S1 File), and the two-parameter Arrhenius equation is therefore used to fit activation energies (*E*_a_) and pre-exponential factors (*A*) for our subsequent kinetic modelling (S3 Table in S1 File).

**Fig 3:**
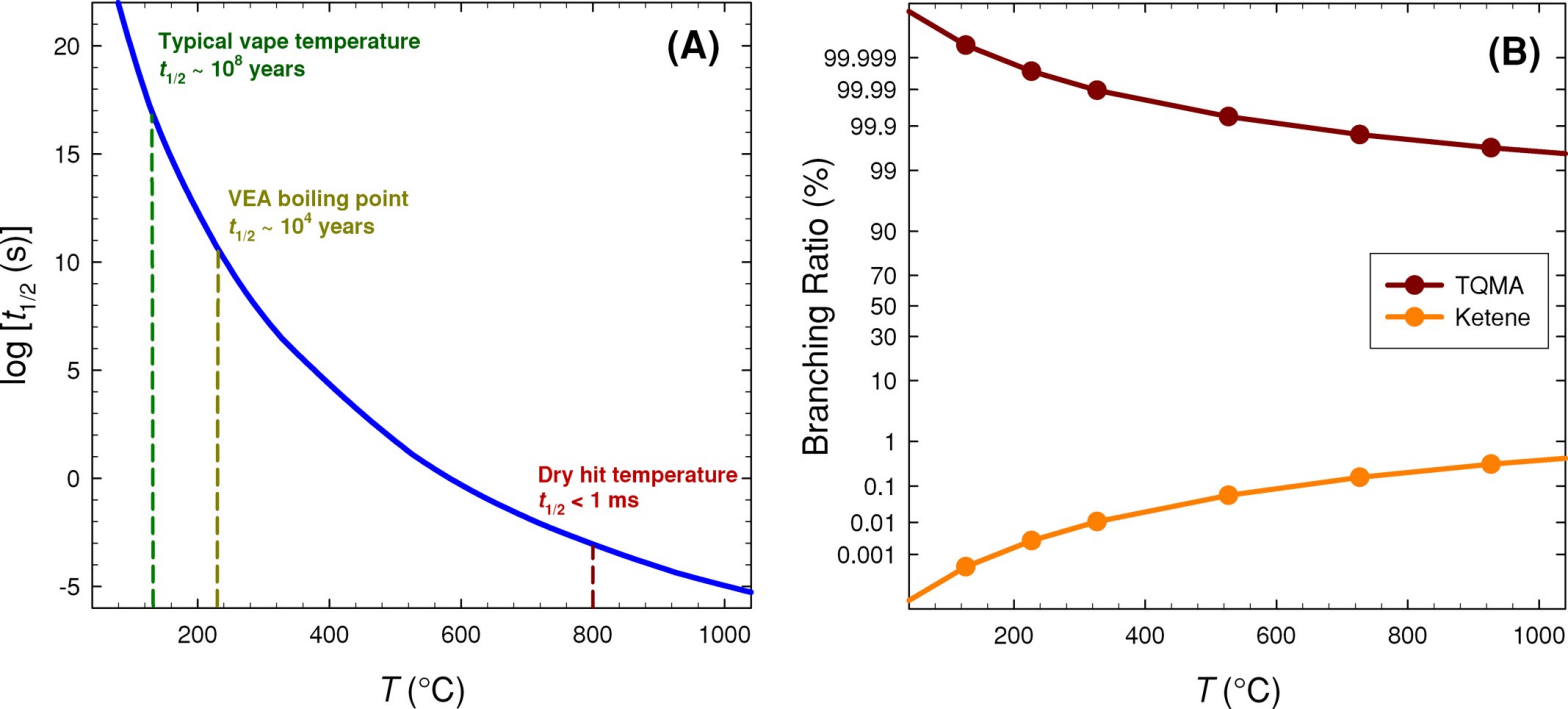
(A) Calculated half-lives of VEA-MC (*t*_1/2_, s) at vaping temperatures, and (B) branching fractions to the different products identified in Fig 2.

Predicted half-lives (*t*_1/2_) of VEA-MC are plotted in Fig 3A, at temperatures relevant to the vaping process. Corresponding product branching fractions are included as Fig 3B. We see from Fig 3A that VEA-MC is thermally stable at typical e-cigarette operating temperatures; at 170°C the lifetime is on the order of 10^8^ years whereas at around the VEA boiling point the lifetime is *ca*. 10^4^ years. It is therefore apparent that under normal operation, vaping pyrolysis of VEA is predicted to be insignificant. However, at the highest temperatures found during vaping, such as during a dry hit, we predict that VEA will rapidly decompose. Peak dry hit temperature is a strong function of working voltage/power, but it can (for instance) reach temperatures of 800°C after 5 puffs at a working voltage of 6 V [13]. From Fig 3B we see that at these temperatures VEA-MC decomposition will largely produce TQMA (isobutylene), with less than 1% yield of ketene.

**Fig 4:**
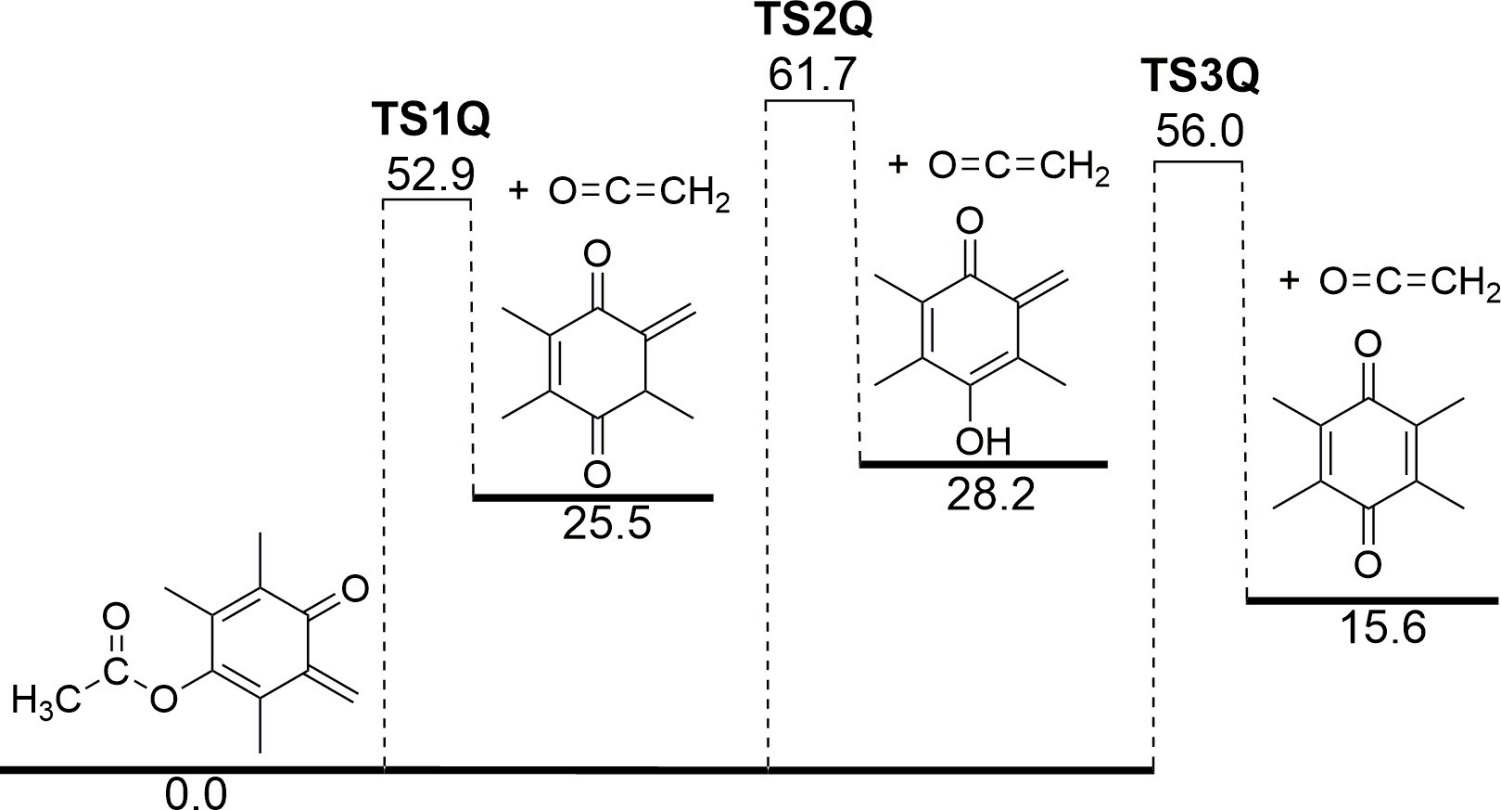
Potential energy diagram for the vaping pyrolysis of trimethyl quinone methide acetate (TQMA). Energies (0 K enthalpies, kcal mol^-1^) are calculated at the DSD-PBEB95-D3(BJ)/def2-TZVPP level of theory.

It is apparent that TQMA will be the primary pyrolysis product of VEA, and its further decomposition therefore needs to be investigated. A potential energy diagram for TQMA decomposition is shown in Fig 4, where we see that, similarly to VEA, TQMA can eliminate ketene through 3 parallel reaction channels. The lowest energy channel involves a 1,4-hydrogen shift from the acetate group to the ring, and proceeds via **TS1Q** with a barrier height of 52.9 kcal/mol. Importantly, this is substantially lower than the barrier for TQMA formation in VEA-MC, and in the pyrolysis of VEA the secondary release of ketene is therefore expected to be rapid (rate coefficients are provided in the S1 File). In the other two channels, 1,3- and 1,7-H shifts to an oxygen atom (**TS2Q**) and to the methylene group (**TS3Q**) transpire, with respective barrier heights of 61.7 and 56.0 kcal/mol. Note also that the quinone methide moiety itself can decompose via ejection of CO, although this requires substantially higher barriers and will be uncompetitive with ketene formation [19, 20].

Vaporization in an e-cigarette conventionally occurs as liquid is drawn through a wick that is heated by an electric element. To simulate vaping pyrolysis, we modelled the gas-phase component of this process as a plug flow reactor (PFR) with internal diameter of 2 mm and length of 20 mm. A two-stage kinetic model is used, where VEA initially decomposes to TQMA, which then releases ketene (S5 Table in S1 File). The inlet flow is assumed to be a vapor mixture of 10% VEA, 50% glycerol, and 40% propylene glycol on a mass basis. The reactor working pressure was fixed at 1 atm and working temperature was varied in the measured range for e-cigarettes, *i*.*e*., 100–1000°C. Testing has shown that vaping e-liquid consumption is a function of air flow and heating power, but is typically around 6.2 mg/s, or 18.5 mg per puff (for a typical puff of 3 s) [21], the value which we have adopted. This results in PFR residence times of 29–8 ms across the working temperature range, with produced gas volumes of 2.1–7.6 mL/s. During vaping, the vaporized e-liquid is mixed with entrained air, at typical air flows of 18.3 mL/s [21]. In our simulations the PFR reactor outlet stream was mixed with air at this flowrate, diluting the vaping products at ratios up to around 1:3. Finally, we need to consider that a single e-cigarette puff will be diluted substantially within the lungs. Assuming the duration of a puff is 3 s, we estimate that a single puff will deliver around 60 mL of gas to the lungs. To obtain in-lung ketene levels, we therefore diluted 3 s worth of the predicted e-cigarette gas products in 5 L of air (a typical human lung capacity). Although all parameters used in these simulations will vary with device, e-liquid, user, and mode of operation, they are sensible and conservative estimates that can be used to provide insight into the potential for gas-phase ketene formation during vaping of VEA contaminated liquids.

**Fig 5:**
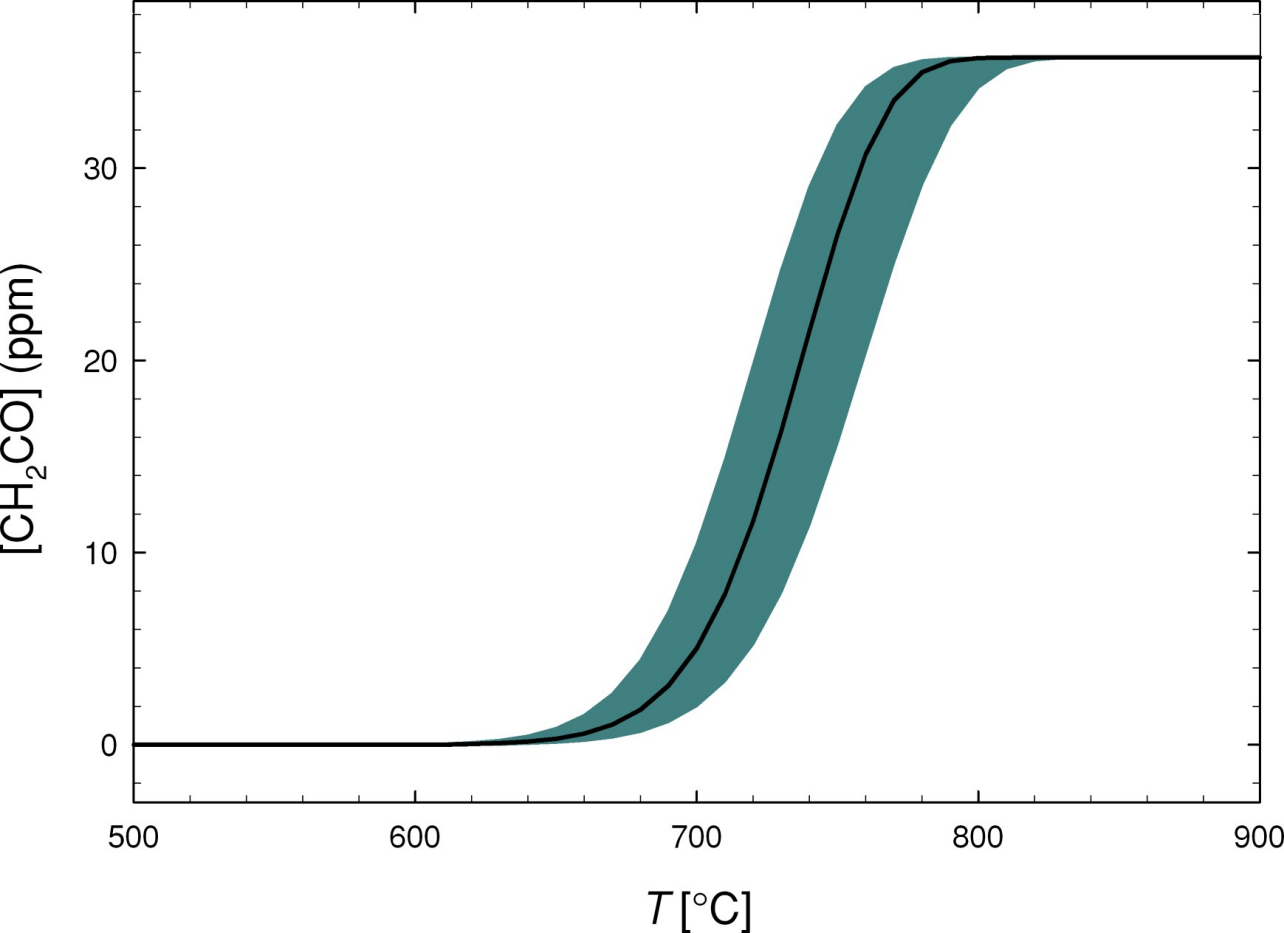
Predicted in-lung ketene concentration ([CH_2_CO], ppm) from a single puff of vitamin E acetate contaminated e-liquid, as a function of temperature. Shaded area shows the uncertainty in predicted concentrations obtained by varying all activation energies by ± 2 kcal/mol.

Predicted in-lung ketene concentrations are plotted in Fig 5, as a function of temperature. At temperatures below 500°C (not shown) no VEA decomposition is predicted to take place. From around 700°C VEA pyrolysis becomes important, delivering several ppm of ketene to the lungs. This increases sharply as temperature increases further, with essentially complete VEA decomposition taking place at around 800°C and above. At these temperatures, lung ketene concentrations are predicted to be above 30 ppm, at which point severe negative health effects would be expected. Note also that, if undiluted in the lung, then ketene concentrations would be around 2800 ppm at these high temperatures.

Our findings demonstrate that at the higher range of e-cigarette operating temperatures, vitamin E acetate decomposition will take place in the gas phase, producing harmful levels of ketene. Under normal e-cigarette operation, however, gas-phase decomposition is negligible. We cannot, however, rule out that catalytic VEA decomposition could produce ketene at these temperatures. The catalysis of VEA decomposition by the heating element and by other compounds in the vapor does however require further investigation.

The results presented here lead us to propose that EVALI is associated with both vitamin E acetate contamination and high e-cigarette operating temperatures. These temperatures are encountered at low liquid levels in poorly controlled vaping devices which do not adjust heating supply for lower liquid delivery rates. In the most extreme cases e-liquids can be vaporized at very high temperatures (> 1000°C), in what users refer to as dry hits. Dry hits have previously been shown to result in e-liquid decomposition to aldehydes [22], and colloquially are known to deliver a pulse of unpleasant burnt gases. Even outside of such extreme conditions, incorrect operation, poor automatic control, or a faulty device can also be responsible for e-liquid vaporization at well above the desired operating temperature.

Clinical and epidemiological information on EVALI is accumulating rapidly. Fortunately, reported cases of this illness have been declining, likely due in part to actions to remove VEA from e-liquids. We are unaware, however, of any data on the correlation of EVALI with vaping conditions. This work highlights a need to track the association of EVALI with vaping conditions (such as the type of device and liquid fill level), in order to correctly assign the causes of this illness and ensure that it does not re-emerge. Experimental studies on the kinetics of VEA pyrolysis are also required, both in the gas phase and on potentially catalytic surfaces such as heating elements. Finally, this work provides a framework for simulating harmful product formation from e-cigarette use and identifies the substantial effect that operating temperature can have on the formation of harmful pyrolysis products. Further studies into the decomposition products of other compounds used in e-liquids appears warranted.

## Supporting information

S1 File (DOCX)
