# Supplementary material for: Does ‘Dry Hit’ vaping of vitamin E acetate contribute to EVALI? Simulating toxic ketene formation during e-cigarette use
Source: PLoS One. 2020 Sep 3;15(9):e0238140. doi: 10.1371/journal.pone.0238140 (PMC7470376; doi:10.1371/journal.pone.0238140)
Supplement: S1 File — (DOCX) [file pone.0238140.s001.docx]

**Does ‘Dry Hit’ Vaping of Vitamin E Acetate contribute to EVALI? Simulating Toxic Ketene Formation during E-cigarette Use**

**Milad Narimani^1^, Gabriel da Silva^1^***

^1^Department of Chemical Engineering, University of Melbourne, Victoria 3010, Australia.

*[gdasilva@unimelb.edu.au](mailto:gdasilva@unimelb.edu.au)

**List of Figures**

[**S1 Fig.** Barrier height alteration of TS4V with carbon chain length of VEA-MC 3](#_Toc45212977)

[**S2 Fig.** Arrhenius plot of calculated VEA-MC thermal decomposition reaction rate coefficients, *k* (s^-1^). Reaction pathways are identified in Fig 2. 4](#_Toc45212978)

**List of Tables**

[**S1 Table.** Energies for vitamin E acetate model compound (VEA-MC) and trimethyl quinone methide acetate (TQMA) transition states, relative to their reactants. Energies are 0 K enthalpies in kcal/mol. 3](#_Toc45213073)

[**S2 Table.** Calculated barrier height (kcal/mol) of **TS4V** for VEA-MC with different carbon chain length 4](#_Toc45213074)

[**S3 Table.** Calculated rate coefficients (*k*, s^-1^) for VEA-MC thermal decomposition 5](#_Toc45213075)

[**S4 Table.** Calculated rate coefficients (*k*, s^-1^) for trimethyl quinone methide acetate thermal decomposition 5](#_Toc45213076)

[**S5 Table.** Derived chemical kinetic model for the vaping pyrolysis of VEA-MC. 5](#_Toc45213077)

[**S6 Table.** VEA-MC and transition states moments of inertia (amu Å^2^) at M06-2X/6-31G(2df,p) level 6](#_Toc45213078)

[**S7 Table.** VEA-MC and transition states vibrational frequencies (cm^-1^) at M06-2X/6-31G(2df,p) level 6](#_Toc45213079)

[**S8 Table.** TQMA and transition states moments of inertia (amu Å^2^) at M06-2X/6-31G(2df,p) level 10](#_Toc45213080)

[**S9 Table.** TQMA and transition states vibrational frequencies (cm^-1^) at M06-2X/6-31G(2df,p) level 10](#_Toc45213081)

[**S10 Table.** Cartesian coordinates (Å) of VEA-MC 13](#_Toc45213082)

[**S11 Table.** Cartesian coordinates (Å) of TS1V 14](#_Toc45213083)

[**S12 Table.** Cartesian coordinates (Å) of TS2V 15](#_Toc45213084)

[**S13 Table.** Cartesian coordinates (Å) of TS3V 16](#_Toc45213085)

[**S14 Table.** Cartesian coordinates (Å) of TS4V 17](#_Toc45213086)

[**S15 Table.** Cartesian coordinates (Å) of TQMA 18](#_Toc45213087)

[**S16 Table.** Cartesian coordinates (Å) of TS1Q 19](#_Toc45213088)

[**S17 Table.** Cartesian coordinates (Å) of TS2Q 20](#_Toc45213089)

[**S18 Table.** Cartesian coordinates (Å) of TS3Q 20](#_Toc45213090)

**S1 Table.** Energies for vitamin E acetate model compound (VEA-MC) and trimethyl quinone methide acetate (TQMA) transition states, relative to their reactants. Energies are 0 K enthalpies in kcal/mol.

| Transition State | M06-2X/6-31G(2df,p) | DSD-PBEB95-D3(BJ)/def2-TZVPP |
| --- | --- | --- |
| TS1V | 66.9 | 66.1 |
| TS2V | 68.7 | 65.5 |
| TS3V | 70.2 | 67.0 |
| TS4V | 61.7 | 57.1 |
| TS1Q | 55.7 | 52.9 |
| TS2Q | 64.8 | 61.7 |
| TS3Q | 58.5 | 56.0 |


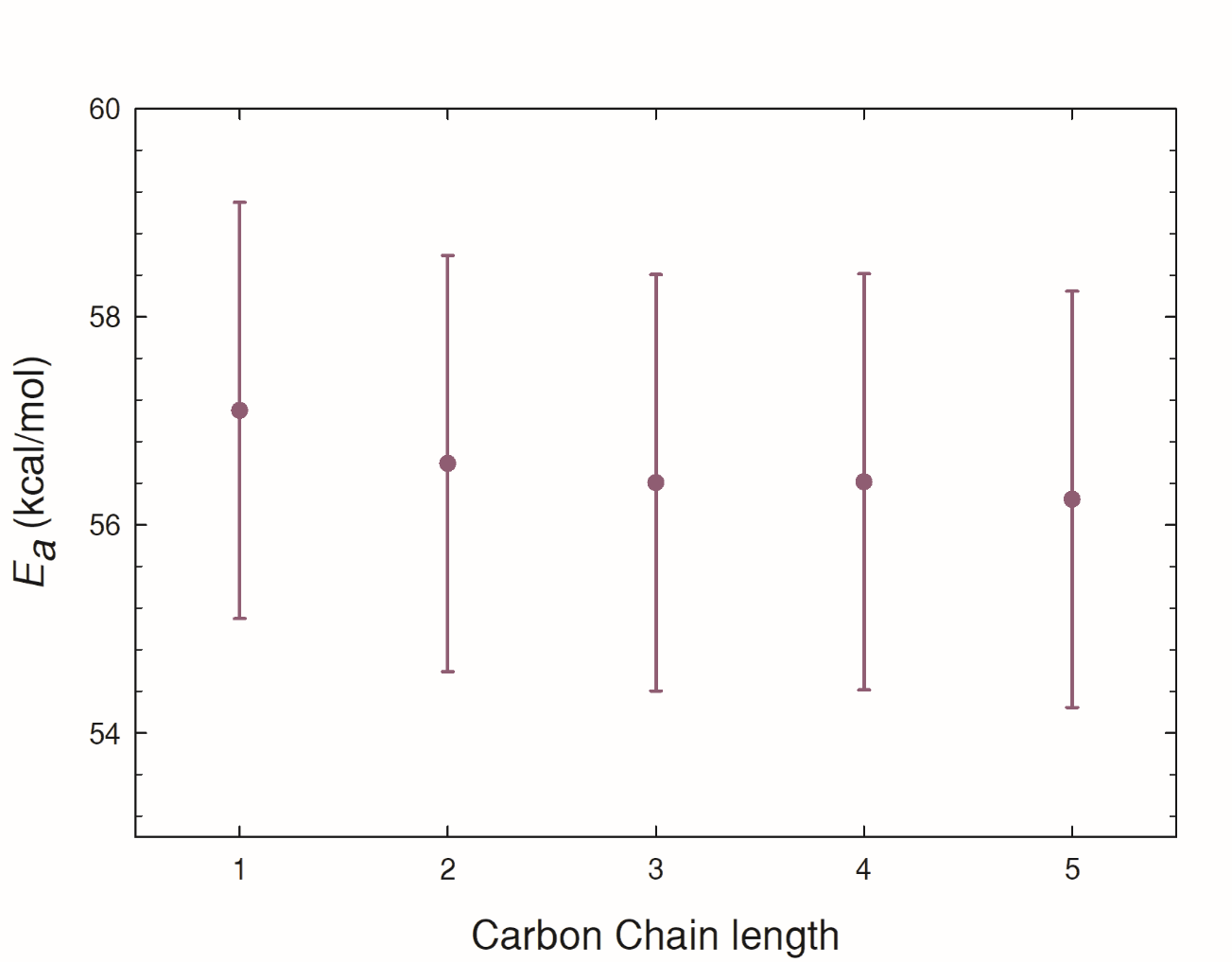


**S1 Fig.** Barrier height alteration of TS4V with carbon chain length of VEA-MC

**S2 Table.** Calculated barrier height (kcal/mol) of **TS4V** for VEA-MC with different carbon chain length

| Carbon Chain length | M06-2X/6-31G(2df,p) | DSD-PBEB95-D3(BJ)/def2-TZVPP |
| --- | --- | --- |
| 1 | 61.74 | 57.09 |
| 2 | 61.44 | 56.59 |
| 3 | 61.22 | 56.40 |
| 4 | 61.18 | 56.41 |
| 5 | 61.13 | 56.24 |
| 8 | 61.16 | - |
| 10 | 61.12 | - |


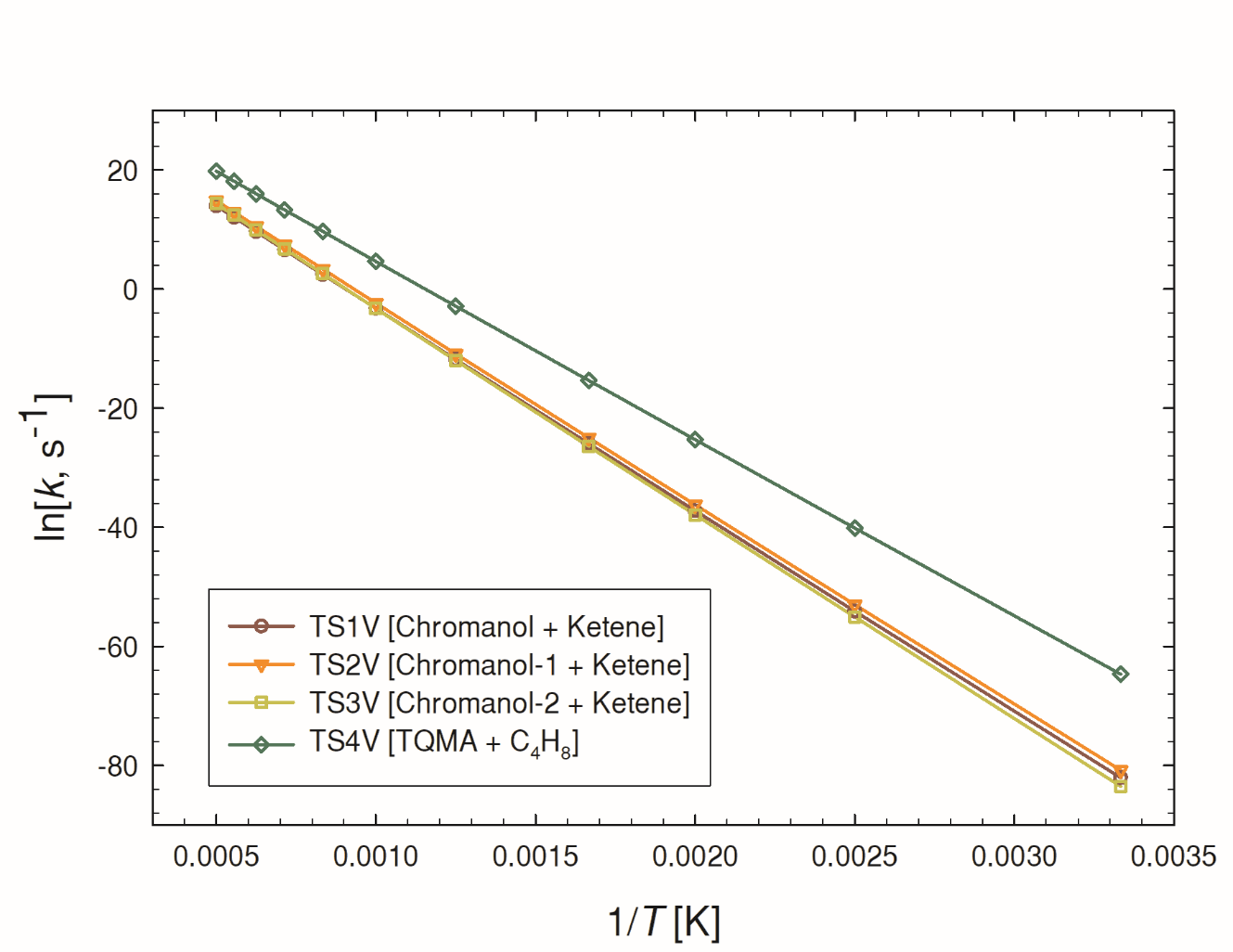


**S2 Fig.** Arrhenius plot of calculated VEA-MC thermal decomposition reaction rate coefficients, *k* (s^-1^). Reaction pathways are identified in Fig 2.

**S3 Table.** Calculated rate coefficients (*k*, s^-1^) for VEA-MC thermal decomposition

| Temperature | TS1V | TS2V | TS3V | TS4V |
| --- | --- | --- | --- | --- |
| 300 | 2.279E-36 | 8.150E-36 | 5.459E-37 | 8.183E-29 |
| 400 | 3.249E-24 | 9.766E-24 | 1.228E-24 | 3.782E-18 |
| 500 | 6.789E-17 | 1.867E-16 | 3.440E-17 | 1.040E-11 |
| 600 | 5.357E-12 | 1.403E-11 | 3.339E-12 | 2.145E-07 |
| 800 | 7.538E-06 | 1.889E-05 | 6.210E-06 | 5.665E-02 |
| 1000 | 3.852E-02 | 9.509E-02 | 3.804E-02 | 1.054E+02 |
| 1200 | 1.171E+01 | 2.879E+01 | 1.314E+01 | 1.620E+04 |
| 1400 | 7.055E+02 | 1.734E+03 | 8.698E+02 | 5.953E+05 |
| 1600 | 1.539E+04 | 3.789E+04 | 2.041E+04 | 8.918E+06 |
| 1800 | 1.702E+05 | 4.200E+05 | 2.393E+05 | 7.336E+07 |
| 2000 | 1.169E+06 | 2.891E+06 | 1.723E+06 | 3.964E+08 |

**S4 Table.** Calculated rate coefficients (*k*, s^-1^) for trimethyl quinone methide acetate thermal decomposition

| Temperature | TS1Q | TS2Q | TS4Q |
| --- | --- | --- | --- |
| 300 | 2.444E-26 | 9.052E-33 | 1.437E-28 |
| 400 | 1.483E-16 | 2.265E-21 | 3.276E-18 |
| 500 | 1.177E-10 | 1.672E-14 | 5.775E-12 |
| 600 | 1.054E-06 | 6.592E-10 | 8.815E-08 |
| 800 | 9.859E-02 | 3.887E-04 | 1.608E-02 |
| 1000 | 9.971E+01 | 1.174E+00 | 2.431E+01 |
| 1200 | 1.033E+04 | 2.510E+02 | 3.295E+03 |
| 1400 | 2.892E+05 | 1.174E+04 | 1.117E+05 |
| 1600 | 3.555E+06 | 2.117E+05 | 1.586E+06 |
| 1800 | 2.519E+07 | 2.019E+06 | 1.258E+07 |
| 2000 | 1.212E+08 | 1.230E+07 | 6.619E+07 |

**S5 Table.** Derived chemical kinetic model for the vaping pyrolysis of VEA-MC.

| Reaction | *A* (s^-1^) | *n* | *E* (cal/mol) |
| --- | --- | --- | --- |
| VEA → TQMA + C_4_H_8_ | 1.04×10^15^ | 0.00 | 59300 |
| TQMA → Trimethyl-2-methylene-1,4-benzoquinone + Ketene | 8.89×10^13^ | 0.00 | 54500 |
| TQMA → Duroquinone + Ketene | 1.08×10^14^ | 0.00 | 57600 |

**Moments of inertia and vibrational frequencies at** **M06-2X/6-31G(2df,p) level of the theory**

S6 Table to S9 Table represents moments of inertia and vibrational frequencies at M06-2X/6-31G(2df,p) level of the theory. All input parameters required for the rate coefficient calculations (Arrhenius plot) are provided in these tables.

**S6 Table.** VEA-MC and transition states moments of inertia (amu Å^2^) at M06-2X/6-31G(2df,p) level

| Ground State |  | Transition State | | | |
| --- | --- | --- | --- | --- | --- |
| VEA |  | TS1V | TS2V | TS3V | TS4V |
| 667.0918 |  | 681.1784 | 747.4901 | 674.7477 | 712.0882 |
| 2094.0532 |  | 2136.2377 | 1997.6591 | 2122.0455 | 2067.5049 |
| 2501.4015 |  | 2573.7394 | 2447.7064 | 2483.5868 | 2448.2498 |

**S7 Table.** VEA-MC and transition states vibrational frequencies (cm^-1^) at M06-2X/6-31G(2df,p) level

| Ground State |  | | Transition State | | | |
| --- | --- | --- | --- | --- | --- | --- |
| VEA | |  | TS1V | TS2V | TS3V | TS4V |
| 38.76 | |  | -1669.14 | -1273.33 | -1240.83 | -400.73 |
| 46.98 | |  | 37.79 | 41.20 | 37.81 | 38.52 |
| 55.85 | |  | 49.54 | 53.67 | 55.69 | 42.62 |
| 80.18 | |  | 69.77 | 71.21 | 68.85 | 58.96 |
| 83.3 | |  | 82.46 | 82.81 | 87.48 | 70.31 |
| 97.2 | |  | 93.82 | 92.99 | 104.16 | 77.72 |
| 127.92 | |  | 106.52 | 102.34 | 120.95 | 93.47 |
| 147.7 | |  | 139.05 | 115.59 | 131.06 | 114.69 |
| 151.73 | |  | 157.76 | 136.33 | 134.04 | 124.20 |
| 167.04 | |  | 166.08 | 164.00 | 142.25 | 136.71 |
| 184.19 | |  | 180.45 | 165.62 | 182.97 | 143.73 |
| 192.87 | |  | 190.88 | 191.41 | 188.57 | 165.43 |
| 214.43 | |  | 206.25 | 205.36 | 203.77 | 179.79 |
| 247.06 | |  | 219.12 | 207.36 | 212.15 | 185.43 |
| 251.1 | |  | 234.48 | 245.43 | 242.86 | 192.42 |
| 279.87 | |  | 251.85 | 263.99 | 257.45 | 198.97 |
| 294.37 | |  | 282.73 | 275.26 | 278.29 | 209.95 |
| 299.93 | |  | 298.02 | 288.35 | 292.10 | 262.73 |
| 314.42 | |  | 309.17 | 303.75 | 297.71 | 278.06 |
| 344.53 | |  | 338.52 | 329.57 | 325.40 | 305.65 |
| 354.48 | |  | 350.59 | 344.42 | 333.34 | 333.79 |
| 379.48 | |  | 356.11 | 352.88 | 348.39 | 349.13 |
| 388.21 | |  | 371.5 | 382.20 | 383.53 | 367.64 |
| 421.01 | |  | 404.97 | 398.99 | 386.65 | 377.38 |
| 437.09 | |  | 423.64 | 409.57 | 407.13 | 393.99 |
| 441.84 | |  | 444.25 | 423.35 | 432.01 | 398.63 |
| 467.12 | |  | 457.64 | 436.18 | 438.70 | 431.04 |
| 491.57 | |  | 475.02 | 463.39 | 457.45 | 431.84 |
| 509.99 | |  | 483.12 | 468.95 | 468.05 | 443.53 |
| 520.32 | |  | 491.54 | 482.78 | 483.76 | 455.58 |
| 563.37 | |  | 513.26 | 496.08 | 498.54 | 500.40 |
| 578.93 | |  | 552.42 | 553.39 | 543.26 | 512.21 |
| 589.8 | |  | 574.14 | 573.04 | 558.23 | 529.14 |
| 619.67 | |  | 590.9 | 591.17 | 586.03 | 579.60 |
| 662.26 | |  | 598.29 | 610.63 | 604.97 | 598.56 |
| 683.81 | |  | 633.91 | 625.74 | 623.32 | 608.23 |
| 717.76 | |  | 680.68 | 630.99 | 641.15 | 658.28 |
| 755.79 | |  | 701.9 | 704.08 | 691.28 | 671.68 |
| 784.44 | |  | 726.28 | 712.56 | 707.81 | 738.76 |
| 823.48 | |  | 758.7 | 761.78 | 761.57 | 814.78 |
| 860.62 | |  | 773.86 | 810.92 | 804.71 | 824.85 |
| 908.63 | |  | 850.05 | 826.80 | 828.54 | 827.43 |
| 927.58 | |  | 866.58 | 850.45 | 856.67 | 845.07 |
| 950.12 | |  | 877.79 | 878.18 | 874.55 | 894.53 |
| 964.76 | |  | 922.18 | 921.18 | 920.21 | 900.38 |
| 977.79 | |  | 945.03 | 945.54 | 943.32 | 921.93 |
| 1006.03 | |  | 958.71 | 958.38 | 951.61 | 949.83 |
| 1015.96 | |  | 974.49 | 969.65 | 975.17 | 965.33 |
| 1018.86 | |  | 1005.13 | 1003.59 | 1002.32 | 987.99 |
| 1035.26 | |  | 1017.41 | 1019.88 | 1017.01 | 1013.36 |
| 1042.74 | |  | 1031.97 | 1026.31 | 1026.12 | 1016.46 |
| 1045.15 | |  | 1037.48 | 1031.53 | 1030.13 | 1032.42 |
| 1056.77 | |  | 1044.18 | 1035.75 | 1039.46 | 1041.44 |
| 1058.97 | |  | 1049.72 | 1039.52 | 1043.18 | 1049.95 |
| 1066.89 | |  | 1053.67 | 1055.59 | 1054.86 | 1054.21 |
| 1069.64 | |  | 1065.41 | 1063.81 | 1057.18 | 1057.09 |
| 1092.72 | |  | 1068.09 | 1066.70 | 1063.79 | 1059.12 |
| 1122.71 | |  | 1074.32 | 1086.94 | 1089.53 | 1065.27 |
| 1157.62 | |  | 1089.35 | 1108.56 | 1098.30 | 1077.79 |
| 1171.93 | |  | 1124.21 | 1125.57 | 1126.67 | 1089.11 |
| 1209.49 | |  | 1156.82 | 1155.10 | 1158.48 | 1117.85 |
| 1258.02 | |  | 1172.69 | 1184.06 | 1187.20 | 1129.71 |
| 1263.94 | |  | 1210.35 | 1204.19 | 1212.50 | 1169.49 |
| 1276.16 | |  | 1260.27 | 1258.60 | 1261.07 | 1188.56 |
| 1290.07 | |  | 1274.74 | 1272.25 | 1273.60 | 1259.32 |
| 1300.23 | |  | 1288.21 | 1275.94 | 1277.91 | 1300.67 |
| 1316.42 | |  | 1300.22 | 1296.25 | 1292.30 | 1325.16 |
| 1367.35 | |  | 1311.98 | 1303.35 | 1306.00 | 1362.73 |
| 1382.74 | |  | 1365.19 | 1320.71 | 1311.18 | 1381.97 |
| 1393.49 | |  | 1381.54 | 1351.30 | 1366.21 | 1393.00 |
| 1399.26 | |  | 1388.1 | 1378.05 | 1382.13 | 1394.72 |
| 1403.01 | |  | 1400.58 | 1390.21 | 1389.25 | 1403.69 |
| 1404.52 | |  | 1401.7 | 1392.20 | 1391.62 | 1404.78 |
| 1412.08 | |  | 1404.54 | 1399.27 | 1396.44 | 1410.48 |
| 1412.57 | |  | 1407.45 | 1401.42 | 1401.25 | 1424.07 |
| 1415.58 | |  | 1411.65 | 1405.64 | 1403.48 | 1428.93 |
| 1459.48 | |  | 1413.84 | 1414.51 | 1414.45 | 1457.98 |
| 1462.91 | |  | 1461.52 | 1423.52 | 1418.52 | 1462.20 |
| 1469.18 | |  | 1467.95 | 1457.19 | 1440.48 | 1464.29 |
| 1473.98 | |  | 1468.88 | 1467.02 | 1462.97 | 1469.83 |
| 1476.73 | |  | 1474.41 | 1471.84 | 1469.52 | 1475.19 |
| 1482.82 | |  | 1481.7 | 1473.49 | 1472.03 | 1476.99 |
| 1484.49 | |  | 1485.09 | 1476.96 | 1476.95 | 1480.14 |
| 1487.65 | |  | 1487.13 | 1477.73 | 1486.00 | 1483.06 |
| 1490.67 | |  | 1490.8 | 1486.50 | 1488.89 | 1489.45 |
| 1495.62 | |  | 1495.29 | 1489.98 | 1490.15 | 1493.47 |
| 1498.25 | |  | 1497.94 | 1495.50 | 1492.58 | 1496.21 |
| 1501.84 | |  | 1499.4 | 1498.08 | 1496.07 | 1502.67 |
| 1504.26 | |  | 1504.86 | 1504.23 | 1501.21 | 1505.20 |
| 1512.87 | |  | 1512.11 | 1508.58 | 1504.44 | 1509.10 |
| 1518.56 | |  | 1517.38 | 1509.29 | 1512.85 | 1606.88 |
| 1519.98 | |  | 1523.31 | 1514.65 | 1514.40 | 1626.16 |
| 1652.86 | |  | 1655.17 | 1600.82 | 1597.79 | 1651.01 |
| 1678.06 | |  | 1668.41 | 1625.88 | 1612.51 | 1706.78 |
| 1915.02 | |  | 1858.23 | 1700.95 | 1694.47 | 1916.35 |
| 3043.53 | |  | 2174.93 | 2217.25 | 2233.68 | 3031.99 |
| 3053.99 | |  | 3037.47 | 3040.43 | 3046.15 | 3047.95 |
| 3057.1 | |  | 3052.39 | 3060.56 | 3050.21 | 3056.02 |
| 3060.69 | |  | 3056.85 | 3061.87 | 3055.94 | 3060.67 |
| 3065.83 | |  | 3058.98 | 3065.24 | 3062.62 | 3062.17 |
| 3067.27 | |  | 3064.12 | 3066.10 | 3064.67 | 3084.13 |
| 3071.62 | |  | 3066.09 | 3070.62 | 3066.37 | 3094.45 |
| 3085.25 | |  | 3070.49 | 3074.02 | 3070.97 | 3114.65 |
| 3096.3 | |  | 3093.15 | 3090.31 | 3097.69 | 3121.62 |
| 3118.05 | |  | 3118.61 | 3122.40 | 3106.69 | 3128.94 |
| 3121.26 | |  | 3120.69 | 3122.53 | 3111.93 | 3129.14 |
| 3123.85 | |  | 3121.97 | 3126.27 | 3120.74 | 3153.79 |
| 3124.02 | |  | 3125.66 | 3133.05 | 3128.09 | 3157.02 |
| 3151.44 | |  | 3130.8 | 3149.73 | 3150.28 | 3164.81 |
| 3156.03 | |  | 3149.75 | 3155.35 | 3153.58 | 3173.42 |
| 3157.98 | |  | 3155.4 | 3156.57 | 3157.36 | 3176.32 |
| 3158.8 | |  | 3156.89 | 3157.23 | 3158.31 | 3177.40 |
| 3159.21 | |  | 3157.57 | 3169.56 | 3165.03 | 3178.98 |
| 3177.23 | |  | 3161.64 | 3170.23 | 3172.77 | 3188.18 |
| 3178.03 | |  | 3177.5 | 3181.77 | 3181.56 | 3210.11 |
| 3197.69 | |  | 3192.44 | 3192.10 | 3193.72 | 3243.95 |
| 3208.91 | |  | 3233.11 | 3268.94 | 3263.93 | 3269.54 |

**S8 Table.** TQMA and transition states moments of inertia (amu Å^2^) at M06-2X/6-31G(2df,p) level

| Ground State |  | Transition State | | |
| --- | --- | --- | --- | --- |
| TQMA |  | TS1Q | TS2Q | TS3Q |
| 544.9287 |  | 571.5066 | 528.3024 | 665.9941 |
| 1035.5263 |  | 1062.9920 | 1181.5238 | 830.9926 |
| 1430.2557 |  | 1427.9613 | 1573.1204 | 1143.5006 |

**S9 Table.** TQMA and transition states vibrational frequencies (cm^-1^) at M06-2X/6-31G(2df,p) level

| Ground State |  | | Transition State | | |
| --- | --- | --- | --- | --- | --- |
| TQMA | |  | TS1Q | TS2Q | TS4Q |
| 38.53 | |  | -1178.10 | -1078.30 | -1366.25 |
| 51.96 | |  | 38.59 | 40.20 | 35.80 |
| 72.35 | |  | 54.32 | 61.06 | 47.94 |
| 77.38 | |  | 56.74 | 66.16 | 58.31 |
| 88.11 | |  | 69.58 | 82.07 | 66.56 |
| 113.03 | |  | 97.38 | 96.73 | 96.75 |
| 139.05 | |  | 108.47 | 108.60 | 113.62 |
| 162.73 | |  | 120.88 | 129.58 | 125.41 |
| 195.39 | |  | 170.39 | 143.46 | 158.20 |
| 201.58 | |  | 192.97 | 160.03 | 199.00 |
| 246.18 | |  | 218.86 | 215.07 | 217.47 |
| 294.69 | |  | 263.37 | 218.18 | 256.72 |
| 326.65 | |  | 306.42 | 307.19 | 296.56 |
| 333.79 | |  | 321.66 | 321.56 | 307.38 |
| 353.86 | |  | 330.04 | 344.13 | 330.46 |
| 363.33 | |  | 345.10 | 359.27 | 365.03 |
| 381.58 | |  | 372.13 | 382.03 | 380.80 |
| 421.7 | |  | 400.16 | 386.85 | 385.96 |
| 424.03 | |  | 422.02 | 419.24 | 419.03 |
| 434.23 | |  | 431.90 | 427.62 | 437.76 |
| 497.43 | |  | 450.19 | 438.52 | 438.49 |
| 509.28 | |  | 452.67 | 444.87 | 451.82 |
| 560.71 | |  | 480.77 | 466.08 | 475.71 |
| 577.46 | |  | 528.58 | 517.55 | 489.40 |
| 592.58 | |  | 548.15 | 550.50 | 565.38 |
| 645.61 | |  | 574.32 | 565.67 | 592.14 |
| 663.78 | |  | 635.37 | 630.60 | 628.99 |
| 718.52 | |  | 653.09 | 678.29 | 644.02 |
| 752.91 | |  | 687.01 | 708.40 | 678.28 |
| 812.83 | |  | 736.50 | 722.17 | 748.31 |
| 830.3 | |  | 794.16 | 751.96 | 771.69 |
| 890.34 | |  | 840.91 | 826.21 | 834.40 |
| 908.73 | |  | 847.49 | 842.35 | 836.65 |
| 987.07 | |  | 886.27 | 898.83 | 893.80 |
| 1014.99 | |  | 911.22 | 966.68 | 903.98 |
| 1019.36 | |  | 978.17 | 968.12 | 984.19 |
| 1028.17 | |  | 987.89 | 978.78 | 993.72 |
| 1046 | |  | 1021.14 | 1023.07 | 1014.18 |
| 1053.98 | |  | 1034.32 | 1026.24 | 1025.40 |
| 1058.51 | |  | 1041.55 | 1041.80 | 1030.49 |
| 1061.28 | |  | 1056.50 | 1049.69 | 1051.27 |
| 1064.5 | |  | 1063.69 | 1059.57 | 1062.02 |
| 1115.84 | |  | 1068.98 | 1063.48 | 1062.95 |
| 1155.43 | |  | 1086.44 | 1076.99 | 1111.89 |
| 1193 | |  | 1118.89 | 1114.67 | 1119.32 |
| 1248.78 | |  | 1150.41 | 1151.40 | 1152.95 |
| 1306 | |  | 1231.86 | 1221.75 | 1225.05 |
| 1341.81 | |  | 1314.71 | 1324.16 | 1309.35 |
| 1387.73 | |  | 1355.71 | 1357.34 | 1361.38 |
| 1394.57 | |  | 1379.30 | 1379.88 | 1366.86 |
| 1402.59 | |  | 1390.23 | 1385.35 | 1375.71 |
| 1413.95 | |  | 1393.13 | 1398.28 | 1392.12 |
| 1419.97 | |  | 1396.03 | 1407.88 | 1400.41 |
| 1437.39 | |  | 1400.34 | 1427.44 | 1407.43 |
| 1460.48 | |  | 1432.47 | 1441.74 | 1420.93 |
| 1471.99 | |  | 1446.56 | 1446.81 | 1454.37 |
| 1475.12 | |  | 1463.87 | 1471.99 | 1469.08 |
| 1479.8 | |  | 1478.31 | 1474.81 | 1470.97 |
| 1486.44 | |  | 1482.95 | 1493.15 | 1489.24 |
| 1493.49 | |  | 1490.27 | 1495.91 | 1493.45 |
| 1495.54 | |  | 1500.98 | 1505.61 | 1496.94 |
| 1506.51 | |  | 1504.63 | 1506.26 | 1504.31 |
| 1683.47 | |  | 1509.36 | 1637.06 | 1510.28 |
| 1691.28 | |  | 1645.64 | 1675.25 | 1641.93 |
| 1747.15 | |  | 1693.76 | 1729.01 | 1749.17 |
| 1804.88 | |  | 1726.47 | 1795.42 | 1782.61 |
| 1924.6 | |  | 1808.28 | 2104.17 | 1809.99 |
| 3059.22 | |  | 2255.87 | 2349.78 | 2144.79 |
| 3064.29 | |  | 3047.11 | 2982.51 | 3053.36 |
| 3067.23 | |  | 3058.54 | 3052.74 | 3071.11 |
| 3083.19 | |  | 3062.78 | 3060.35 | 3078.27 |
| 3121.26 | |  | 3109.89 | 3062.19 | 3110.10 |
| 3129.61 | |  | 3117.56 | 3104.61 | 3134.82 |
| 3131.92 | |  | 3126.38 | 3124.10 | 3138.53 |
| 3156.67 | |  | 3141.11 | 3136.13 | 3140.70 |
| 3181.03 | |  | 3163.90 | 3152.90 | 3172.00 |
| 3185.73 | |  | 3189.40 | 3181.87 | 3174.63 |
| 3190.48 | |  | 3189.76 | 3185.23 | 3187.34 |
| 3194.63 | |  | 3191.66 | 3191.62 | 3189.57 |
| 3209.52 | |  | 3233.23 | 3194.89 | 3227.80 |
| 3287.19 | |  | 3285.13 | 3286.16 | 3267.83 |

**Cartesian Coordinates (Angstrom) at M06-2X/6-31G(2df,p)** **level of the theory**

S10 Table to S18 Table represents Cartesian coordinates of the optimized geometries of VEA-MC, TQMA and their decomposition reactions transition states. All these structures are obtained at M06-2X/6-31G(2df,p) level of the theory.

**S10 Table.** Cartesian coordinates (Å) of VEA-MC

|  |  |  |  |
| --- | --- | --- | --- |
| C | 1.16838800 | 1.31925700 | -0.30373700 |
| C | -0.17702100 | 1.57133500 | -0.03923000 |
| C | -1.06561400 | 0.49325600 | 0.06687400 |
| C | -0.63045600 | -0.82679100 | -0.07146400 |
| C | 0.71807300 | -1.07784600 | -0.34772400 |
| C | 1.59091000 | -0.00200900 | -0.44876700 |
| C | -1.60317700 | -1.97052900 | 0.08447300 |
| C | -2.90755200 | -1.49829000 | 0.71683000 |
| C | -3.36508100 | -0.18128700 | 0.09247700 |
| H | -1.80158900 | -2.42810000 | -0.89359600 |
| H | -3.69147200 | -2.25320900 | 0.60902700 |
| O | -2.36349400 | 0.81214400 | 0.33287100 |
| H | -2.76002600 | -1.32346000 | 1.78803500 |
| H | -1.15529500 | -2.75951900 | 0.69661100 |
| C | 3.84176000 | -0.43432100 | 0.22495400 |
| C | 3.34187600 | -0.35177400 | 1.64701400 |
| H | 4.18724900 | -0.52070700 | 2.30956500 |
| H | 2.56443600 | -1.09827300 | 1.82479700 |
| O | 2.92169100 | -0.24828100 | -0.76145200 |
| O | 4.97429900 | -0.64818300 | -0.08170600 |
| H | 2.89687300 | 0.62689500 | 1.84093700 |
| C | 2.14629900 | 2.45681200 | -0.42984100 |
| H | 2.33310900 | 2.92794000 | 0.54211300 |
| H | 3.09918100 | 2.11779800 | -0.83491500 |
| H | 1.75251300 | 3.23682300 | -1.08677200 |
| C | -0.67016900 | 2.98434500 | 0.12370500 |
| H | -0.62945000 | 3.52595600 | -0.82762400 |
| H | -1.69831400 | 2.99955000 | 0.47838500 |
| H | -0.04664500 | 3.53734500 | 0.83202700 |
| C | 1.21360200 | -2.48929800 | -0.51955700 |
| H | 2.21019500 | -2.50415900 | -0.95964700 |
| H | 1.25649900 | -3.01726900 | 0.44040800 |
| H | 0.54471900 | -3.06279600 | -1.16662200 |
| C | -4.61188900 | 0.34570800 | 0.78449100 |
| H | -4.87381100 | 1.32931800 | 0.38753300 |
| H | -5.45202400 | -0.33361800 | 0.62050200 |
| H | -4.43795200 | 0.43969800 | 1.85891300 |
| C | -3.59115300 | -0.30235500 | -1.41443900 |
| H | -4.32277300 | -1.08610300 | -1.62931700 |
| H | -3.96675700 | 0.64478500 | -1.80837200 |
| H | -2.66142100 | -0.54162100 | -1.93604900 |

**S11 Table.** Cartesian coordinates (Å) of TS1V

|  |  |  |  |
| --- | --- | --- | --- |
| C | -1.19821800 | 1.19542600 | 0.35802400 |
| C | 0.12007900 | 1.51744200 | 0.04823800 |
| C | 1.06498600 | 0.49135100 | -0.08273600 |
| C | 0.70970400 | -0.84412100 | 0.09795600 |
| C | -0.61529600 | -1.16825500 | 0.42013900 |
| C | -1.56523100 | -0.14879600 | 0.52094600 |
| C | 1.74523600 | -1.93251900 | -0.05985100 |
| C | 2.99722600 | -1.40338600 | -0.75020300 |
| C | 3.39714100 | -0.04489300 | -0.17629200 |
| H | 2.00660000 | -2.34757300 | 0.92269500 |
| H | 3.82739500 | -2.10890000 | -0.65180600 |
| O | 2.33497200 | 0.88066500 | -0.40921400 |
| H | 2.80286700 | -1.26573900 | -1.81943200 |
| H | 1.32751700 | -2.76598600 | -0.63339600 |
| C | -4.10878600 | -0.21088400 | -0.63145900 |
| C | -3.79264900 | -1.50697000 | -1.07132500 |
| H | -4.56428500 | -1.99354900 | -1.65901900 |
| H | -3.30183100 | -1.50418600 | 0.19071100 |
| O | -2.86865900 | -0.45346000 | 0.78246100 |
| O | -4.76812600 | 0.72291000 | -0.51766400 |
| H | -2.78682400 | -1.55107300 | -1.49041700 |
| C | -2.22743900 | 2.27995200 | 0.54207800 |
| H | -2.58857700 | 2.66579000 | -0.41813300 |
| H | -3.08966200 | 1.90283700 | 1.09175100 |
| H | -1.80556300 | 3.12684500 | 1.08876300 |
| C | 0.53270500 | 2.95346300 | -0.14414900 |
| H | 1.52555000 | 3.01657900 | -0.58458400 |
| H | -0.17382700 | 3.48006900 | -0.79145500 |
| H | 0.55178900 | 3.48884800 | 0.81167000 |
| C | -0.99107100 | -2.60380200 | 0.69406400 |
| H | -1.90872400 | -2.66338000 | 1.28103200 |
| H | -1.14238200 | -3.18063500 | -0.22590900 |
| H | -0.20485400 | -3.10966900 | 1.26001700 |
| C | 4.59019700 | 0.53074400 | -0.92277200 |
| H | 4.80716100 | 1.53882500 | -0.56147400 |
| H | 5.47296900 | -0.09487400 | -0.76857600 |
| H | 4.37702800 | 0.58413800 | -1.99297500 |
| C | 3.67937100 | -0.11266700 | 1.32502400 |
| H | 4.45709700 | -0.85190800 | 1.53645700 |
| H | 4.01773800 | 0.86329800 | 1.68076200 |
| H | 2.77960200 | -0.38319600 | 1.88256500 |

**S12 Table.** Cartesian coordinates (Å) of TS2V

|  |  |  |  |
| --- | --- | --- | --- |
| C | 1.03916000 | 1.70189800 | -0.25115100 |
| C | -0.30058600 | 1.70862400 | 0.03209100 |
| C | -1.04809200 | 0.48493800 | 0.09146700 |
| C | -0.46371400 | -0.72334000 | -0.13683100 |
| C | 0.95517000 | -0.78121700 | -0.41236600 |
| C | 1.70790500 | 0.45227100 | -0.54428500 |
| C | -1.27043500 | -1.99201700 | -0.06489200 |
| C | -2.61210300 | -1.73635300 | 0.61533700 |
| C | -3.24622500 | -0.44122100 | 0.10358700 |
| H | -1.42179100 | -2.40046300 | -1.07294500 |
| H | -3.30056000 | -2.57137500 | 0.45713700 |
| O | -2.37073300 | 0.65188000 | 0.39400200 |
| H | -2.46355700 | -1.62889700 | 1.69526300 |
| H | -0.70992600 | -2.75725100 | 0.48262200 |
| C | 3.89483600 | -0.85951200 | 0.71027700 |
| C | 2.87893900 | -0.88102100 | 1.62673800 |
| H | 2.91947800 | -1.72974000 | 2.29947400 |
| H | 1.65481700 | -0.90620700 | 0.73212500 |
| O | 2.95867700 | 0.38988100 | -0.70821200 |
| O | 4.86640800 | -1.11957600 | 0.15822500 |
| H | 2.63022200 | 0.08451700 | 2.05427800 |
| C | 1.87216900 | 2.95558500 | -0.30834700 |
| H | 1.80029500 | 3.52848500 | 0.62060200 |
| H | 2.91375700 | 2.68222300 | -0.47249800 |
| H | 1.55725300 | 3.61490400 | -1.12307400 |
| C | -1.06455300 | 2.97494100 | 0.29948000 |
| H | -1.50398000 | 2.95128800 | 1.30089200 |
| H | -0.43549800 | 3.85836100 | 0.20765500 |
| H | -1.90306400 | 3.05942900 | -0.39704000 |
| C | 1.50498100 | -2.01257200 | -1.11712000 |
| H | 2.53064600 | -1.81661900 | -1.43256300 |
| H | 1.50032400 | -2.89792900 | -0.47363600 |
| H | 0.91228100 | -2.24531800 | -2.00645700 |
| C | -3.49818900 | -0.48448600 | -1.40370200 |
| H | -4.12801400 | -1.34028600 | -1.66206800 |
| H | -4.00435300 | 0.43054500 | -1.72023500 |
| H | -2.55928600 | -0.56294000 | -1.95672900 |
| C | -4.52931400 | -0.12986500 | 0.85700500 |
| H | -5.28276100 | -0.89586100 | 0.65732600 |
| H | -4.33821400 | -0.09384000 | 1.93207200 |
| H | -4.92258700 | 0.83958100 | 0.54194000 |

**S13 Table.** Cartesian coordinates (Å) of TS3V

|  |  |  |  |
| --- | --- | --- | --- |
| C | 1.34991800 | 0.93873800 | -0.41563900 |
| C | 0.00608700 | 1.39235300 | -0.14786500 |
| C | -0.98074100 | 0.47295600 | 0.07335000 |
| C | -0.71993300 | -0.93038700 | -0.03394500 |
| C | 0.52997500 | -1.39881600 | -0.35516700 |
| C | 1.61421900 | -0.47958200 | -0.59990000 |
| C | -1.84684900 | -1.91291300 | 0.18691500 |
| C | -3.08642100 | -1.26066800 | 0.79017500 |
| C | -3.34702900 | 0.10317300 | 0.15384200 |
| H | -2.09472700 | -2.37786400 | -0.77633400 |
| H | -3.96074000 | -1.90718900 | 0.67111200 |
| O | -2.22178700 | 0.93973200 | 0.42475000 |
| H | -2.93939300 | -1.09727800 | 1.86312000 |
| H | -1.50071000 | -2.73206700 | 0.82458500 |
| C | 4.09146600 | -0.10271600 | 0.81811200 |
| C | 3.08795200 | 0.25495600 | 1.67395600 |
| H | 3.37684000 | 1.02230000 | 2.38326700 |
| H | 2.50125900 | -0.57660100 | 2.05111800 |
| O | 2.80171100 | -0.87109400 | -0.75946500 |
| O | 5.10775200 | -0.22217000 | 0.30410300 |
| H | 2.01110200 | 0.74226700 | 0.75245800 |
| C | 2.33690300 | 1.91302700 | -1.03663600 |
| H | 2.55597000 | 2.75675900 | -0.37547200 |
| H | 3.26535900 | 1.38837800 | -1.26488400 |
| H | 1.94126500 | 2.31917800 | -1.97227000 |
| C | -0.27264600 | 2.86654000 | -0.07886600 |
| H | 0.32550300 | 3.34030000 | 0.70741700 |
| H | -0.00277400 | 3.35896900 | -1.01850300 |
| H | -1.32450800 | 3.05325400 | 0.12700500 |
| C | 0.81849300 | -2.86849900 | -0.48686000 |
| H | 0.17119800 | -3.34475300 | -1.23059700 |
| H | 1.85751400 | -3.00410700 | -0.78425300 |
| H | 0.65639600 | -3.39670100 | 0.45953900 |
| C | -4.52068600 | 0.79906000 | 0.82474100 |
| H | -4.64520700 | 1.80439900 | 0.41542500 |
| H | -5.44229100 | 0.23567100 | 0.65870700 |
| H | -4.34558900 | 0.88173000 | 1.89991000 |
| C | -3.56664900 | 0.00999400 | -1.35640000 |
| H | -4.40948500 | -0.64961500 | -1.58061200 |
| H | -3.78260100 | 1.00276600 | -1.75834600 |
| H | -2.68013900 | -0.37483400 | -1.86635700 |

**S14 Table.** Cartesian coordinates (Å) of TS4V

|  |  |  |  |
| --- | --- | --- | --- |
| C | -1.08777200 | 1.29766200 | 0.18467600 |
| C | 0.13757000 | 1.64246000 | -0.30935300 |
| C | 1.06073400 | 0.59172000 | -0.72259400 |
| C | 0.63212400 | -0.78936200 | -0.58305900 |
| C | -0.63191900 | -1.11811800 | -0.00140400 |
| C | -1.45531600 | -0.08497400 | 0.32490300 |
| C | 1.60095100 | -1.72554900 | -0.93657400 |
| C | 3.07513600 | -1.75621600 | 0.50781500 |
| C | 3.55222200 | -0.46856400 | 0.58882200 |
| H | 1.37671000 | -2.78631100 | -0.88686400 |
| H | 2.42029100 | -2.12265300 | 1.29204700 |
| O | 2.22566700 | 0.82685600 | -1.09425600 |
| H | 3.66670800 | -2.50702700 | -0.00654900 |
| H | 2.32064900 | -1.41820900 | -1.68529800 |
| C | -3.80106600 | -0.46955700 | 0.09802100 |
| C | -3.57555400 | -0.43028200 | -1.39478200 |
| H | -4.54631600 | -0.48574600 | -1.88144200 |
| H | -2.95343500 | -1.27442900 | -1.70234300 |
| O | -2.69941000 | -0.33419000 | 0.88877200 |
| O | -4.86301400 | -0.62647300 | 0.61690600 |
| H | -3.04876300 | 0.47937700 | -1.69182200 |
| C | -2.09944100 | 2.31969000 | 0.62935800 |
| H | -1.69313900 | 3.32905700 | 0.59171600 |
| H | -2.99370800 | 2.29278900 | -0.00404400 |
| H | -2.43746700 | 2.10761700 | 1.64661300 |
| C | 0.61024300 | 3.06395600 | -0.44886500 |
| H | 1.61636500 | 3.06161800 | -0.86753800 |
| H | -0.04010500 | 3.64631200 | -1.10814700 |
| H | 0.63774400 | 3.57600500 | 0.51804700 |
| C | -1.03592800 | -2.55226200 | 0.19663000 |
| H | -1.98335800 | -2.62462100 | 0.73004300 |
| H | -1.13408200 | -3.07270900 | -0.76213600 |
| H | -0.28064100 | -3.09122200 | 0.77770400 |
| C | 4.75143400 | -0.03181300 | -0.18880800 |
| H | 5.63866800 | -0.06388100 | 0.45632500 |
| H | 4.93227500 | -0.69183800 | -1.04016900 |
| H | 4.62608400 | 0.98806400 | -0.55192200 |
| C | 2.96218600 | 0.45784000 | 1.60072700 |
| H | 3.18940400 | 1.50131600 | 1.37924600 |
| H | 1.87100400 | 0.33242800 | 1.62974300 |
| H | 3.33554100 | 0.21474200 | 2.60233600 |

**S15 Table.** Cartesian coordinates (Å) of TQMA

|  |  |  |  |
| --- | --- | --- | --- |
| C | -0.19800000 | 1.18013700 | -0.30952400 |
| C | -1.51758900 | 1.21121600 | -0.02585400 |
| C | -2.25818200 | -0.06179900 | 0.13566500 |
| C | -1.48740900 | -1.34657300 | -0.03507800 |
| C | -0.05851900 | -1.30210600 | -0.33652100 |
| C | 0.51725300 | -0.09355600 | -0.44383700 |
| C | -2.16705100 | -2.48905800 | 0.11443800 |
| H | -1.69895700 | -3.46101400 | 0.01521300 |
| O | -3.44389400 | -0.07936200 | 0.39277000 |
| H | -3.22532900 | -2.44689600 | 0.34422500 |
| C | 2.80592200 | 0.09946700 | 0.21587300 |
| C | 2.32496100 | -0.07282100 | 1.63598900 |
| H | 3.17295400 | 0.08291600 | 2.29839400 |
| H | 1.92205300 | -1.07915200 | 1.77479400 |
| O | 1.85692300 | 0.01963200 | -0.76582400 |
| O | 3.93918000 | 0.27809600 | -0.10180300 |
| H | 1.52276500 | 0.63067000 | 1.87172000 |
| C | 0.61905000 | 2.42392200 | -0.51865000 |
| H | 1.39258800 | 2.52362600 | 0.25145900 |
| H | 1.14501500 | 2.37527000 | -1.47541200 |
| H | -0.00045700 | 3.31828100 | -0.49511500 |
| C | -2.31576700 | 2.47599600 | 0.13014900 |
| H | -2.35671200 | 3.03967800 | -0.80661200 |
| H | -3.33393400 | 2.22287200 | 0.42214100 |
| H | -1.88482700 | 3.13151300 | 0.89137800 |
| C | 0.70776900 | -2.58019700 | -0.50384700 |
| H | 1.74103200 | -2.38645700 | -0.78931600 |
| H | 0.70073500 | -3.16423500 | 0.42274900 |
| H | 0.25077800 | -3.20176600 | -1.27957100 |

**S16 Table.** Cartesian coordinates (Å) of TS1Q

|  |  |  |  |
| --- | --- | --- | --- |
| C | -0.64237900 | 1.31540000 | -0.34822300 |
| C | -1.84749900 | 0.85832300 | 0.04128100 |
| C | -2.08780600 | -0.59565000 | 0.24303700 |
| C | -0.97969100 | -1.54294000 | -0.12014200 |
| C | 0.34578100 | -1.00255900 | -0.42777000 |
| C | 0.50479700 | 0.39611300 | -0.61745000 |
| C | -1.28682700 | -2.84498000 | -0.14974900 |
| H | -0.56178700 | -3.60357300 | -0.41895500 |
| O | -3.15063300 | -0.99445400 | 0.66987700 |
| H | -2.29259100 | -3.15942100 | 0.10025500 |
| C | 3.07833600 | 0.26652900 | 0.75834600 |
| C | 2.13545200 | -0.29011800 | 1.59708200 |
| H | 2.53842800 | -1.12457200 | 2.16474000 |
| H | 1.13805000 | -0.72183700 | 0.79148800 |
| O | 1.64234700 | 0.88933300 | -0.80447200 |
| O | 4.03628800 | 0.60395400 | 0.24155700 |
| H | 1.61845900 | 0.46532300 | 2.18641300 |
| C | -0.38131900 | 2.78360000 | -0.52757500 |
| H | -0.73751100 | 3.35396300 | 0.33496900 |
| H | 0.68271100 | 2.96131300 | -0.67213600 |
| H | -0.91934600 | 3.16325500 | -1.40304400 |
| C | -3.00147500 | 1.77635200 | 0.32284900 |
| H | -3.15191900 | 2.47987000 | -0.50061800 |
| H | -3.90947500 | 1.19566000 | 0.47737900 |
| H | -2.81773700 | 2.37166600 | 1.22404400 |
| C | 1.37552100 | -1.93116900 | -1.03127500 |
| H | 2.24989800 | -1.35946200 | -1.34638200 |
| H | 1.69450900 | -2.70706700 | -0.32558700 |
| H | 0.96695200 | -2.43918100 | -1.91072100 |

**S17 Table.** Cartesian coordinates (Å) of TS2Q

|  |  |  |  |
| --- | --- | --- | --- |
| C | 0.81627100 | -1.27045000 | -0.24938100 |
| C | 2.03992900 | -0.77123400 | 0.01494300 |
| C | 2.22439500 | 0.69355000 | 0.13233900 |
| C | 1.00755600 | 1.56869200 | -0.04402100 |
| C | -0.27219700 | 0.95511600 | -0.35513200 |
| C | -0.38221700 | -0.40672400 | -0.42139600 |
| C | 1.19802200 | 2.88403700 | 0.13744600 |
| H | 0.39382700 | 3.60566000 | 0.06120000 |
| O | 3.31598700 | 1.17362400 | 0.36604100 |
| H | 2.18979200 | 3.24585300 | 0.37822900 |
| C | -3.46253500 | -0.16871400 | 0.44348200 |
| C | -2.66276000 | -0.57354600 | 1.51884800 |
| H | -3.24651300 | -0.84503400 | 2.39613200 |
| H | -1.86645300 | 0.16476900 | 1.68574300 |
| O | -1.52190100 | -1.03282100 | -0.58679000 |
| O | -4.24020300 | 0.06351100 | -0.33751500 |
| H | -2.04762600 | -1.32193700 | 0.81522000 |
| C | 0.56469800 | -2.74377400 | -0.39624400 |
| H | 0.03288300 | -3.13239400 | 0.48003800 |
| H | -0.08684900 | -2.92595400 | -1.25253500 |
| H | 1.49117400 | -3.30560400 | -0.50856000 |
| C | 3.27070000 | -1.61751200 | 0.19992200 |
| H | 3.55868500 | -2.11332700 | -0.73262400 |
| H | 4.09797100 | -0.98378800 | 0.51687200 |
| H | 3.11359300 | -2.39703900 | 0.95012000 |
| C | -1.46307000 | 1.81882500 | -0.65908100 |
| H | -2.14800600 | 1.28983500 | -1.32482100 |
| H | -2.02590000 | 2.13558100 | 0.23544300 |
| H | -1.16038500 | 2.73926400 | -1.16468900 |

**S18 Table.** Cartesian coordinates (Å) of TS3Q

|  |  |  |  |
| --- | --- | --- | --- |
| C | -1.56335700 | -0.82189100 | 0.10501600 |
| C | -1.84916400 | 0.47845900 | -0.06265800 |
| C | -0.79723800 | 1.50682300 | 0.19099800 |
| C | 0.53029000 | 1.07562800 | 0.75446900 |
| C | 0.83377400 | -0.27349500 | 0.82726800 |
| C | -0.20946200 | -1.27215700 | 0.56624000 |
| C | 1.51870200 | 2.05853200 | 0.85549400 |
| H | 2.37279600 | 1.88974000 | 1.50308000 |
| O | -1.00304500 | 2.67487200 | -0.05676800 |
| H | 1.22243100 | 3.08974300 | 0.70431300 |
| C | 1.88289300 | -0.46121700 | -1.33588700 |
| C | 2.45991600 | 0.79443900 | -1.45094600 |
| H | 2.00644300 | 1.37422400 | -2.25297800 |
| H | 3.54522300 | 0.75429200 | -1.48802600 |
| O | 0.01038100 | -2.46416800 | 0.66780700 |
| O | 1.63621700 | -1.55834300 | -1.59860800 |
| H | 2.11814600 | 1.54342500 | -0.37774000 |
| C | -2.52832300 | -1.93721100 | -0.15220500 |
| H | -2.64770100 | -2.54174300 | 0.75076100 |
| H | -3.50228700 | -1.57219500 | -0.47452500 |
| H | -2.12264600 | -2.61079000 | -0.91186100 |
| C | -3.18231200 | 0.99271800 | -0.53387400 |
| H | -3.97938400 | 0.70076600 | 0.15511200 |
| H | -3.15625600 | 2.07909200 | -0.59997200 |
| H | -3.43562800 | 0.58922500 | -1.51799200 |
| C | 2.09910100 | -0.77409800 | 1.47728900 |
| H | 2.20979300 | -1.84186000 | 1.28630100 |
| H | 2.98799200 | -0.24839800 | 1.11471200 |
| H | 2.06374600 | -0.62359700 | 2.56214600 |
